# Supplementary material for: HOPX hypermethylation promotes metastasis via activating SNAIL transcription in nasopharyngeal carcinoma
Source: Nat Commun. 2017 Feb 1;8:14053. doi: 10.1038/ncomms14053 (PMC5296651; doi:10.1038/ncomms14053)

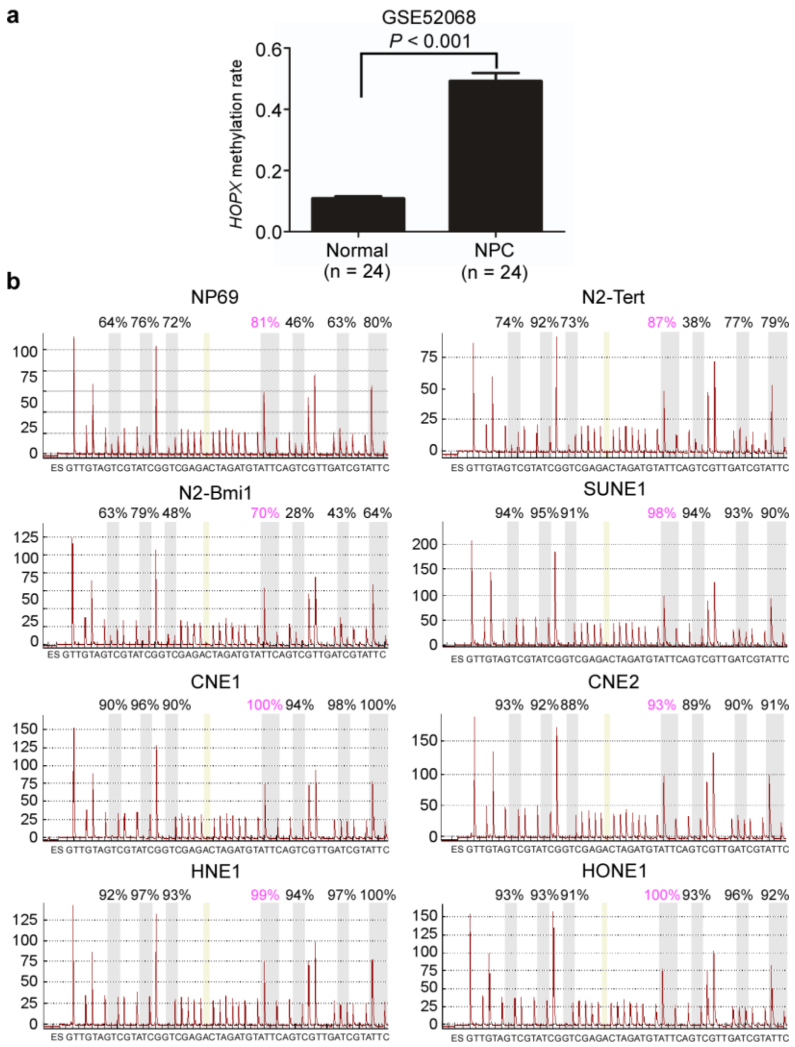

Supplementary Figure 1. *HOPX* is hypermethylated in NPC. (a) Methylation levels of *HOPX* in Normal (n = 24) and NPC (n = 24) tissues from the genome-wide methylation microarray data. Mean ± s.d.; Student's *t*-tests. **(b)** Bisulfite pyrosequencing analysis of the *HOPX* promoter region in NPEC (NP69, N2-Tert and N2-Bmi1) and NPC (SUNE1, CNE1, CNE2, HNE1 and HONE1) cell lines. Magenta words: CG site of cg21899596. These data are representative of three independent experiments.

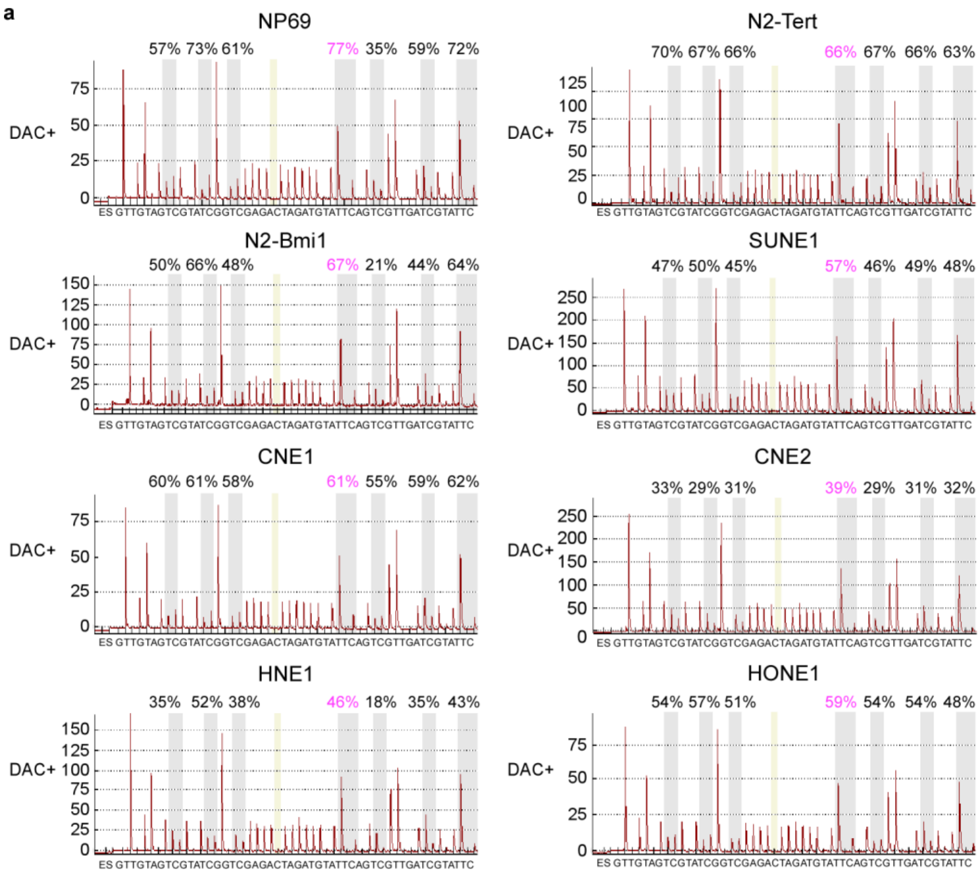

Supplementary Figure 2. *HOPX* is hypermethylated in NPC cells. (**a**) Bisulfite pyrosequencing analysis of the *HOPX* promoter region in NPEC (NP69, N2-Tert and N2-Bmi1) and NPC (SUNE1, CNE1, CNE2, HNE1 and HONE1) cell lines following treatment with DAC. Magenta words: CG site of cg21899596. These data are representative of three independent experiments.

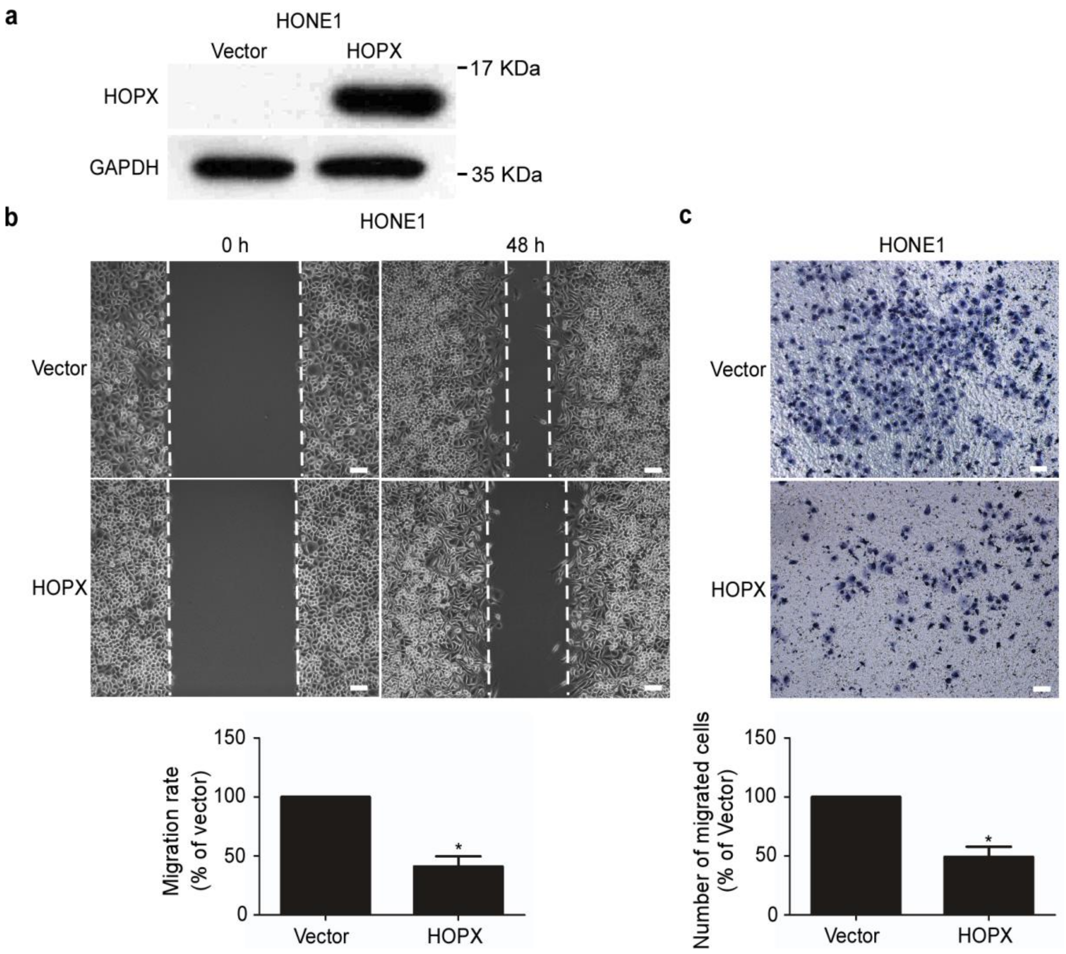

Supplementary Figure 3. HOPX suppresses HONE1 cell migration *in vitro*. (a) The ectopic expression of HOPX in HONE1 cells was confirmed by western blotting. **(b,c)** Migration ability was measured using a wound healing assay (200 ×) **(b)** and Transwell assay without Matrigel (200 ×) **(c)** in HONE1 cells with the vector or HOPX overexpression. Scale bar: 100 μm; Mean ± s.d.; *, $P < 0.01$ compared with vector; Student's *t*-tests. These data are representative of three independent experiments.

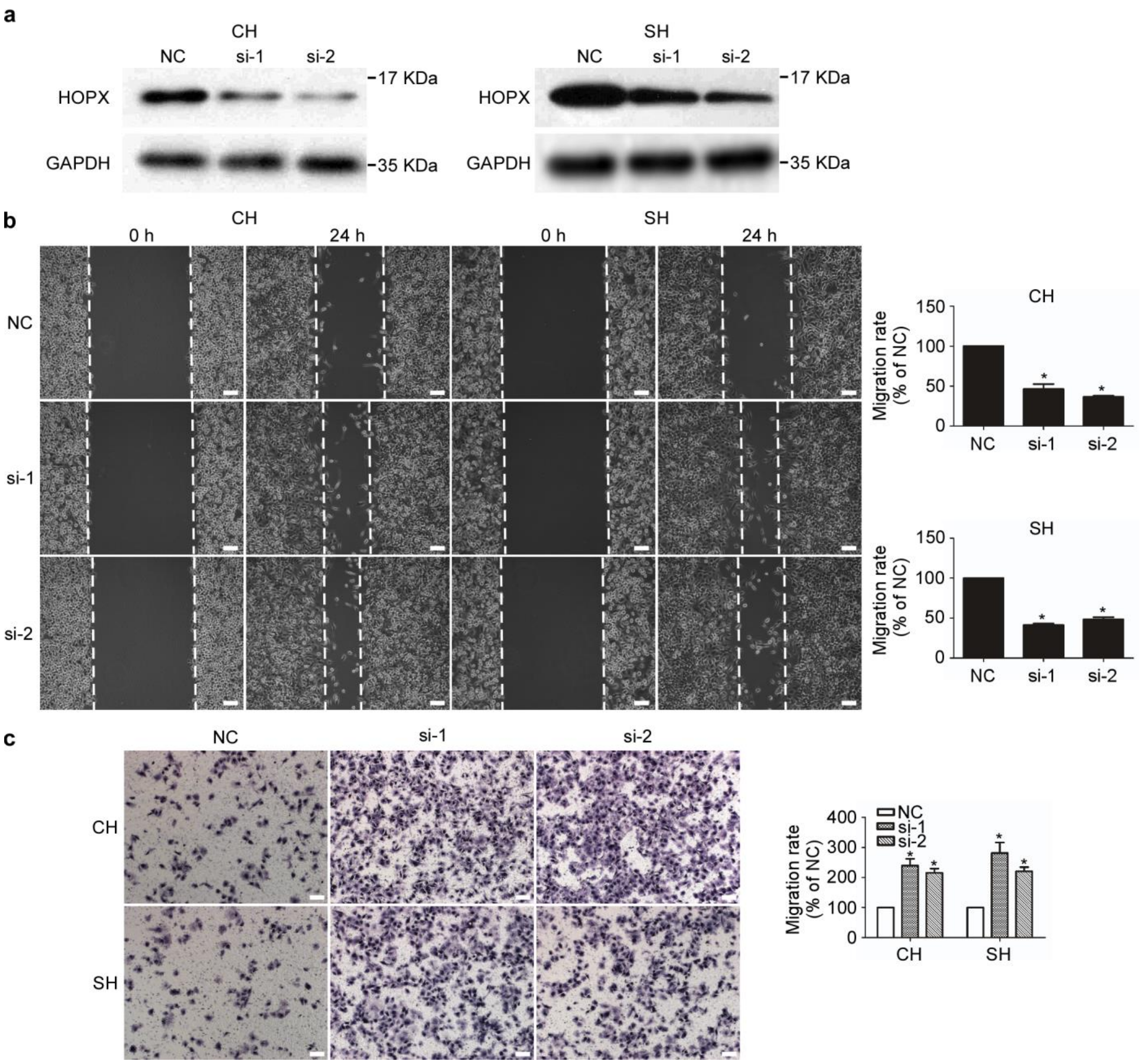

Supplementary Figure 4. Silencing HOPX promotes NPC cell migration *in vitro*. Control NC, or *HOPX*-siRNAs (si-1 and si-2) were used to knock down the expression of HOPX in CNE2 and SUNE1 cells with stable HOPX overexpression (CH and SH). **(a)** The silencing effects of HOPX in CH and SH cells were confirmed by western blotting. **(b,c)** Wound healing assay (200 ×) **(b)** and Transwell assay without Matrigel (200 ×) **(c)** were used to measure the migration abilities. Scale bar: 100 μm; mean ± s.d.; *, $P < 0.01$ compared with NC; Student's *t*-tests. These data are representative of three independent experiments.

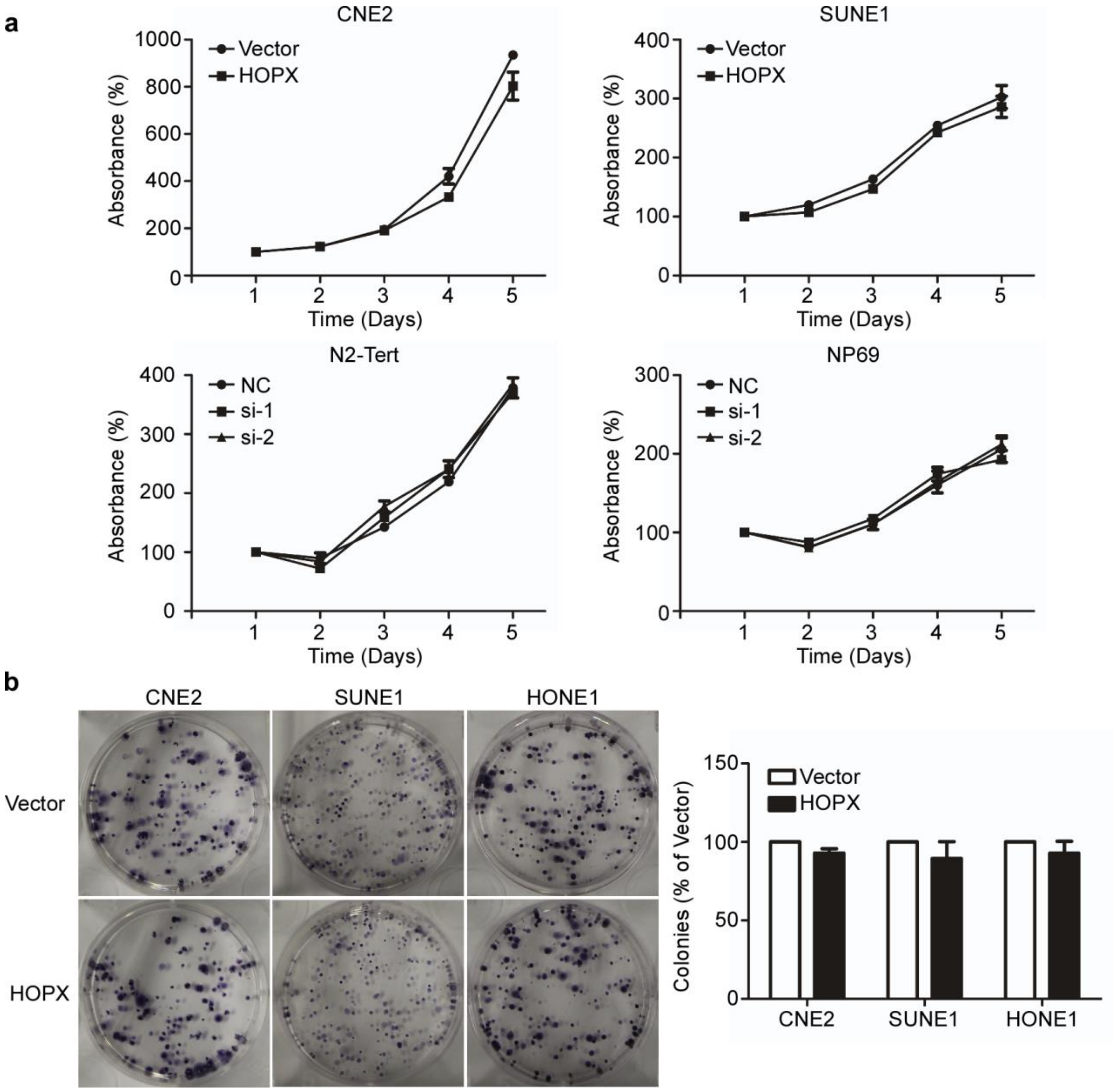

Supplementary Figure 5. HOPX exhibits little effect on NPC cell proliferation *in vitro*. (a) MTT assay was performed to examine the effect of HOPX on cell viability in CNE2 and SUNE1 cells stably overexpressed the vector or HOPX, or N2-Tert and NP69 cells transfected with the control NC or *HOPX*-siRNAs (si-1 and si-2). Mean ± s.d.; $P > 0.05$ compared with vector or NC; Student's *t*-tests. **(b)** Colony formation assay was used to examine the effect of HOPX on colonization in CNE2, SUNE1 and HONE1 cells with the vector or HOPX overexpression. Mean ± s.d.; $P > 0.05$ compared with vector; Student's *t*-tests. These data are representative of three independent experiments.

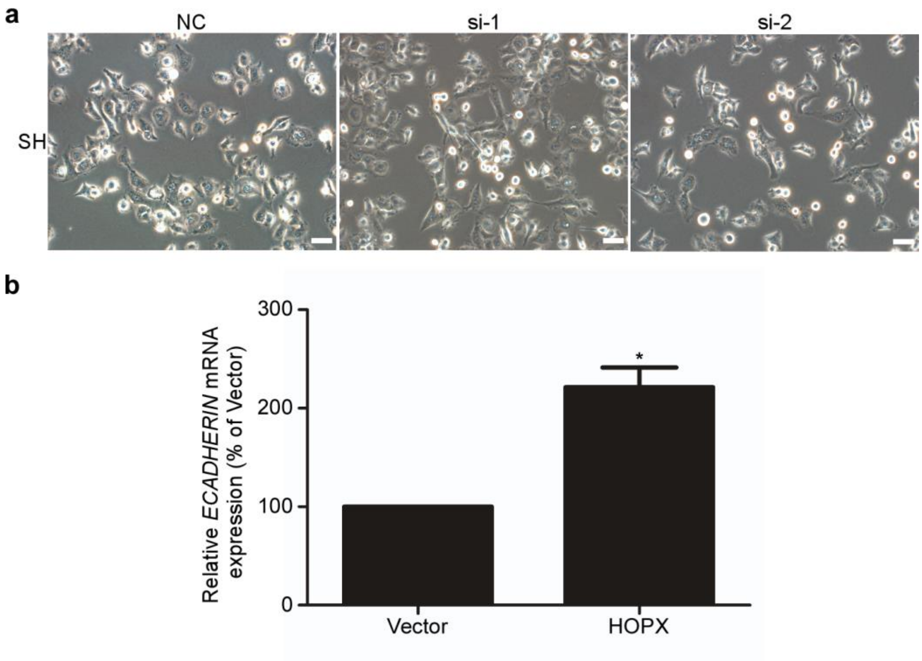

Supplementary Figure 6. HOPX partially inhibits EMT in NPC cells. (a) Phase contrast images (200 ×) of SH cells which were transfected with the control NC or *HOPX*-siRNAs (si-1 and si-2). SH cells indicated SUNE1 cells with HOPX overexpression. Scale bar: 100 μm. **(b)** The mRNA level of *ECADHERIN* was identified using a tumor metastasis PCR array in SUNE1 cells stably overexpressed the vector or HOPX. Mean ± s.d.; *, $P < 0.01$ compared with vector; Student's *t*-tests. These data are representative of three independent experiments.

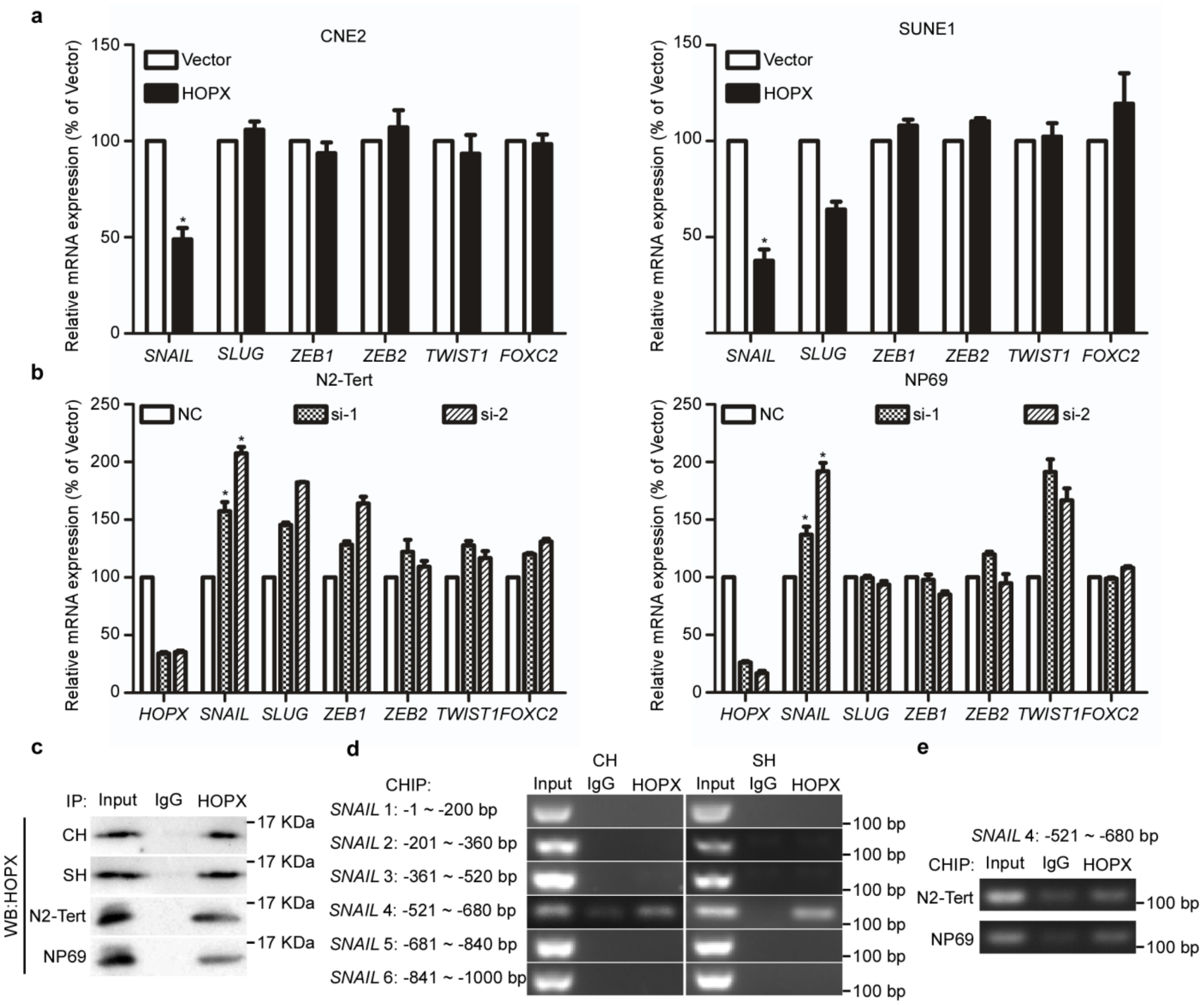

Supplementary Figure 7. HOPX suppresses *SNAIL* expression in NPC. (a,b) Real time RT-PCR was used to examine the mRNA level of EMT-TFs (*SNAIL*, *SLUG*, *ZEB1*, *ZEB2*, *TWIST1* and *FOXC2*) in CNE2 and SUNE1 cells stably overexpressed the vector or HOPX **(a)**, or N2-Tert and NP69 cells transiently expressed control NC or *HOPX*-siRNAs (si-1 and si-2) **(b)**. Mean ± s.d.; *, $P < 0.01$ compared with vector or NC; Student's *t*-tests. **(c)** ChIP assay using an anti-HOPX antibody was performed to pull down HOPX. Western blotting (WB) was conducted to examine HOPX using an anti-HOPX Antibody. **(d,e)** ChIP-PCR assay was conducted to assess the enrichment of HOPX in the promoter regions of *SNAIL* in CH, SH, N2-Tert and NP69 cells. CH and SH cells indicated CNE2 and SUNE1 cells with HOPX exogenous expression. These data are representative of three independent experiments.

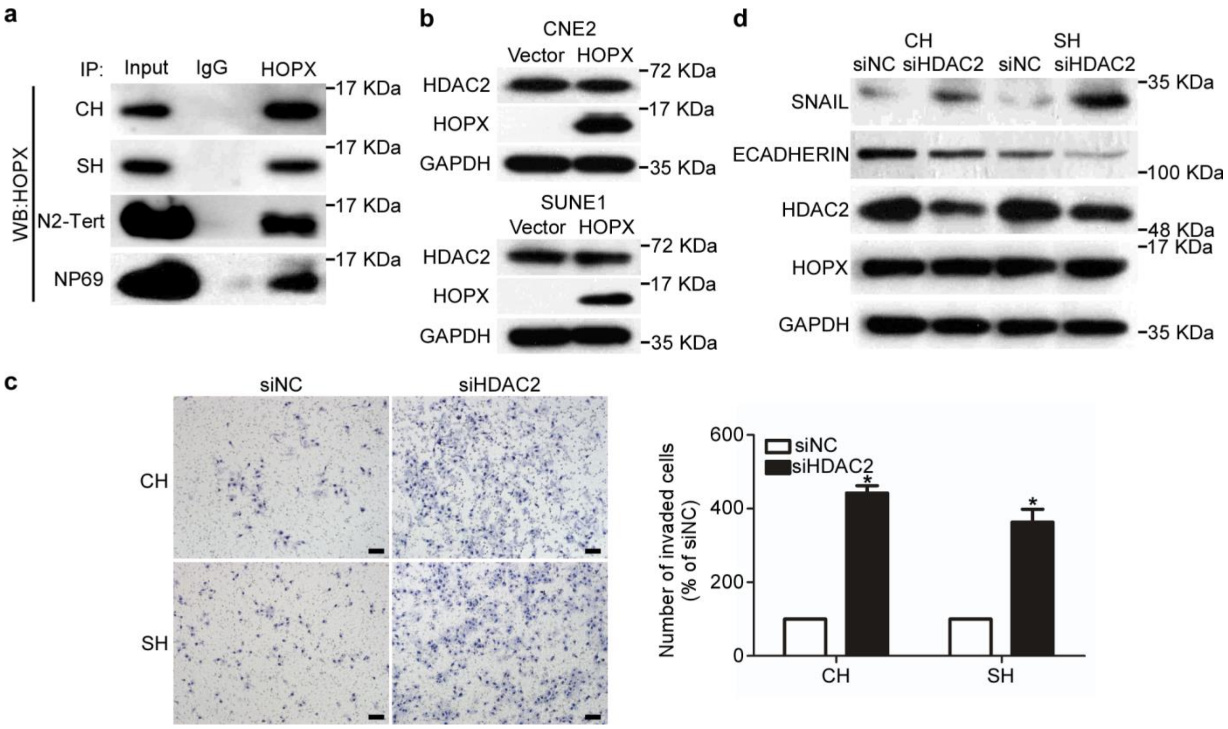

Supplementary Figure 8. Silencing HDAC2 reverses the inhibitory effects of HOPX on invasiveness and EMT in NPC. (a) IP assay using anti-HOPX antibody was performed to pull down HOPX. Western blotting (WB) was conducted to examine HOPX using an anti-HOPX Antibody. **(b)** Western blotting assay was used to assess the expression levels of HDAC2, HOPX and GAPDH. **(c)** Transwell assay with Matrigel (200 ×) was used to measure the invasive ability of CH and SH cells which were transfected with the control siNC or siHDAC2. Scale bar: 100 μm; mean ± s.d.; *, $P < 0.01$ compared with siNC; Student's *t*-tests. **(d)** Western blotting assay was used to examine the expression levels of SNAIL, ECADHERIN, HDAC2, HOPX and GAPDH in CH and SH cells which were transfected with the control siNC or siHDAC2. CH and SH cells indicated CNE2 and SUNE1 cells with HOPX exogenous expression. These data are representative of three independent experiments.

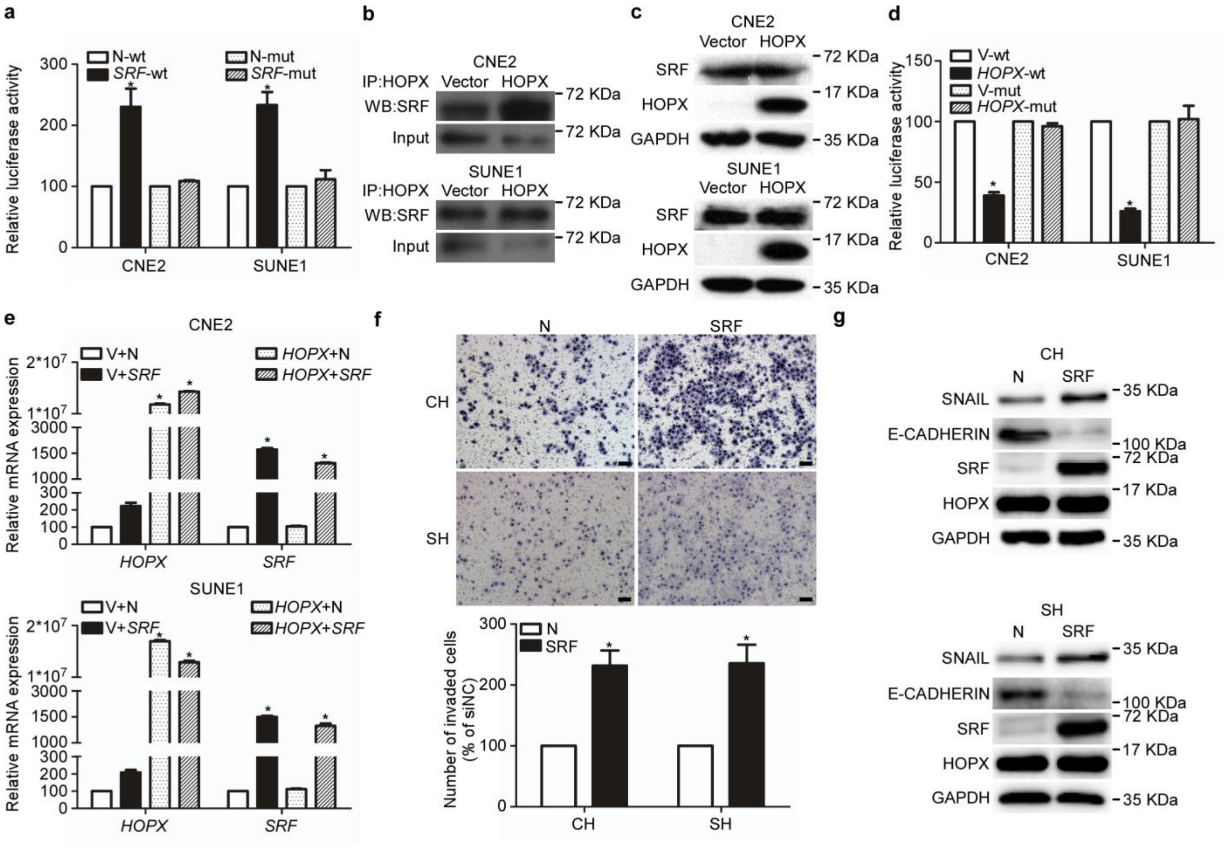

Supplementary Figure 9. HOPX inhibits SRF-mediated *SNAIL* transcription in NPC. (a) Wild type and mutant *SNAIL*-luciferase reporters were constructed and transfected with the control N or *SRF* construct in CNE2 and SUNE1 cells for 24 h. Luciferase reporter assay was used to detect the luciferase activity. Mean ± s.d.; *, $P < 0.01$ compared with N-wt; Student's *t*-tests. **(b)** Co-IP assay was used to measure the interaction between HOPX and SRF in CNE2 and SUNE1 cells stably overexpressed the vector or HOPX. **(c)** Western blotting assay was used to assess the expression levels of SRF, HOPX and GAPDH. **(d)** Wild type and mutant *SNAIL*-luciferase reporters were constructed and transfected with the vector (V) or *HOPX* construct in CNE2 and SUNE1 cells for 24 h. Luciferase reporter assay was used to detect the luciferase activity. Mean ± s.d.; *, $P < 0.01$ compared with V-wt; Student's *t*-tests. **(e)** CNE2 and SUNE1 cells were co-transfected with the *HOPX* and *SRF*. V and N were used as empty vectors of *HOPX* and *SRF*, respectively. Relative *SRF* and *HOPX* mRNA expressions were measured via real time RT-PCR. Mean ± s.d.; *, $P < 0.01$ compared with V + N; Student's *t*-tests. **(f)** Transwell assay with Matrigel (200 ×) was used to measure the invasive ability of CH and SH cells which were transfected with the control N or *SRF* expression plasmids. Scale bar: 100 μm; mean ± s.d.; *, $P < 0.01$ compared with N; Student's *t*-tests. **(g)** Western blotting assay was used to examine the expression levels of SNAIL, ECADHERIN, SRF, HOPX and GAPDH in CH and SH cells which were transfected with the control N or *SRF* expression plasmids. CH and SH cells indicated CNE2 and SUNE1 cells with HOPX exogenous expression. These data are representative of three independent experiments.

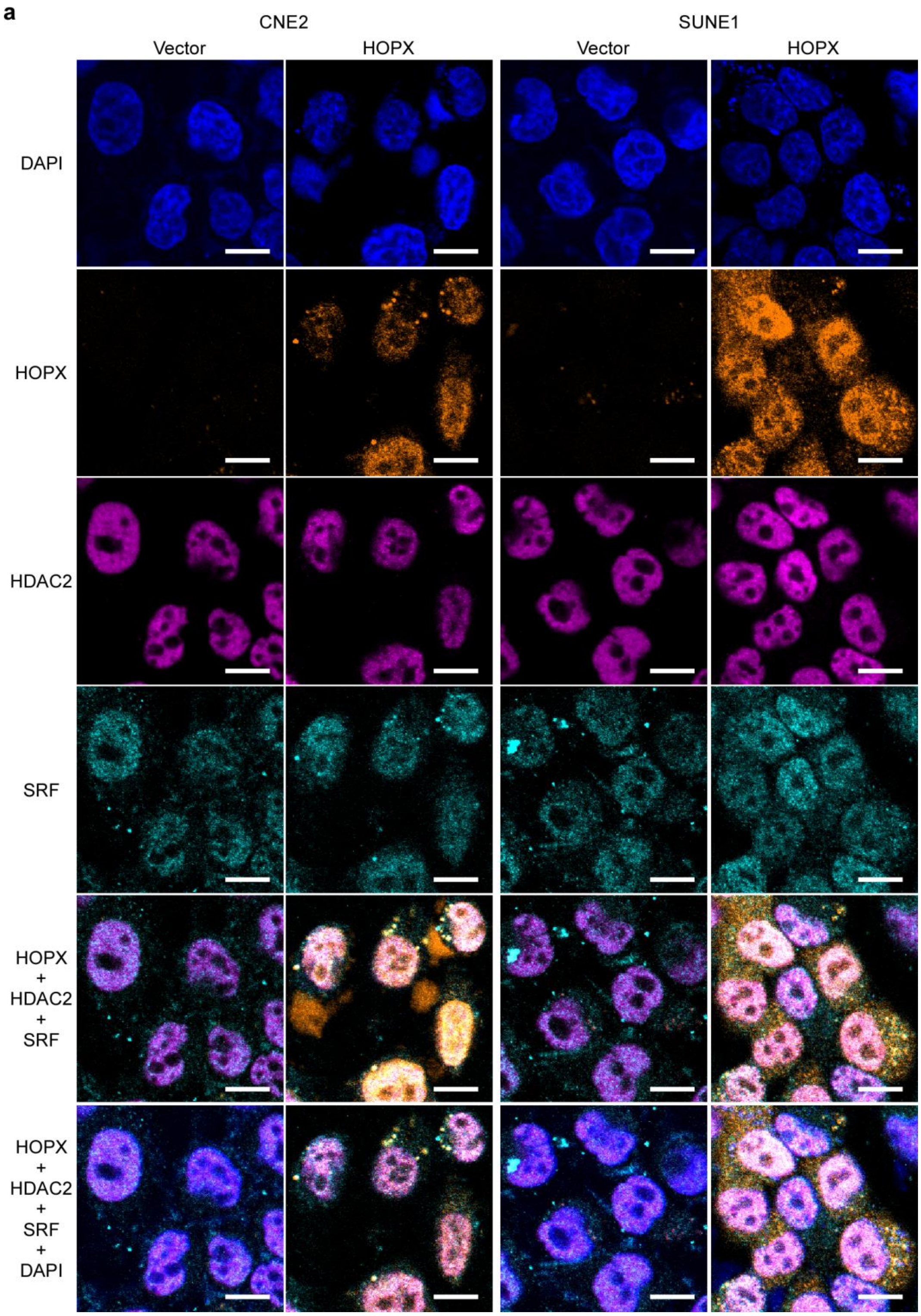

Supplementary Figure 10. HOPX is co-localized with HDAC2 and SRF in NPC cells. (a) Immunofluorescence images (600 ×) of HOPX (orange), HDAC2 (magenta) and SRF (cyan) expression in CNE2 and SUNE1 cells stably overexpressed the vector or HOPX. Scale bar: 100 μm. These data are representative of three independent experiments.

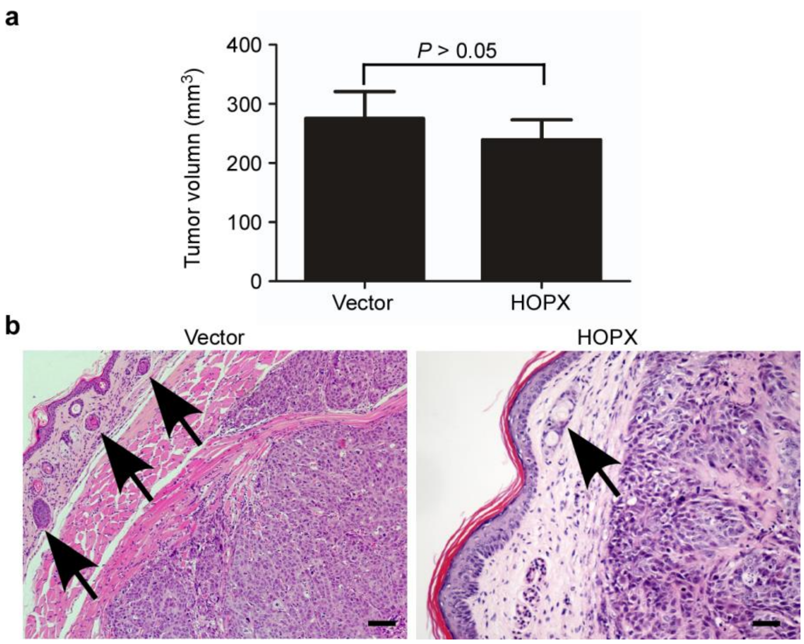

Supplementary Figure 11. Restoring HOPX expression inhibits NPC cell aggressiveness *in vivo*. (a) Quantification of the average volumes of the primary foot pad tumors. Mean ± s.d.; $P > 0.05$ compared with vector; Student's *t*-tests. **(b)** Representative images (200 ×) of the microscopic primary tumor in foot pad stained with H & E. Arrows represent lymphatic vessels. Scale bar: 100 μm. These data are representative of eight independent experiments (each mouse sample was considered as one independent experiment; three technological replications were repeated in each sample).

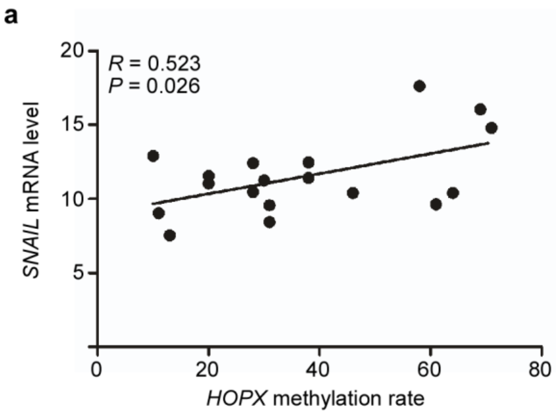

Supplementary Figure 12. The methylation level of *HOPX* is positively associated with the *SNAIL* mRNA level in NPC. (a) *HOPX* methylation levels were determined via bisulfite pyrosequencing, while the *SNAIL* mRNA levels were determined via real time RT-PCR in NPC tissues (n = 24). Statistical analysis was performed using the Pearson's coefficient test. These data are representative of three independent experiments.

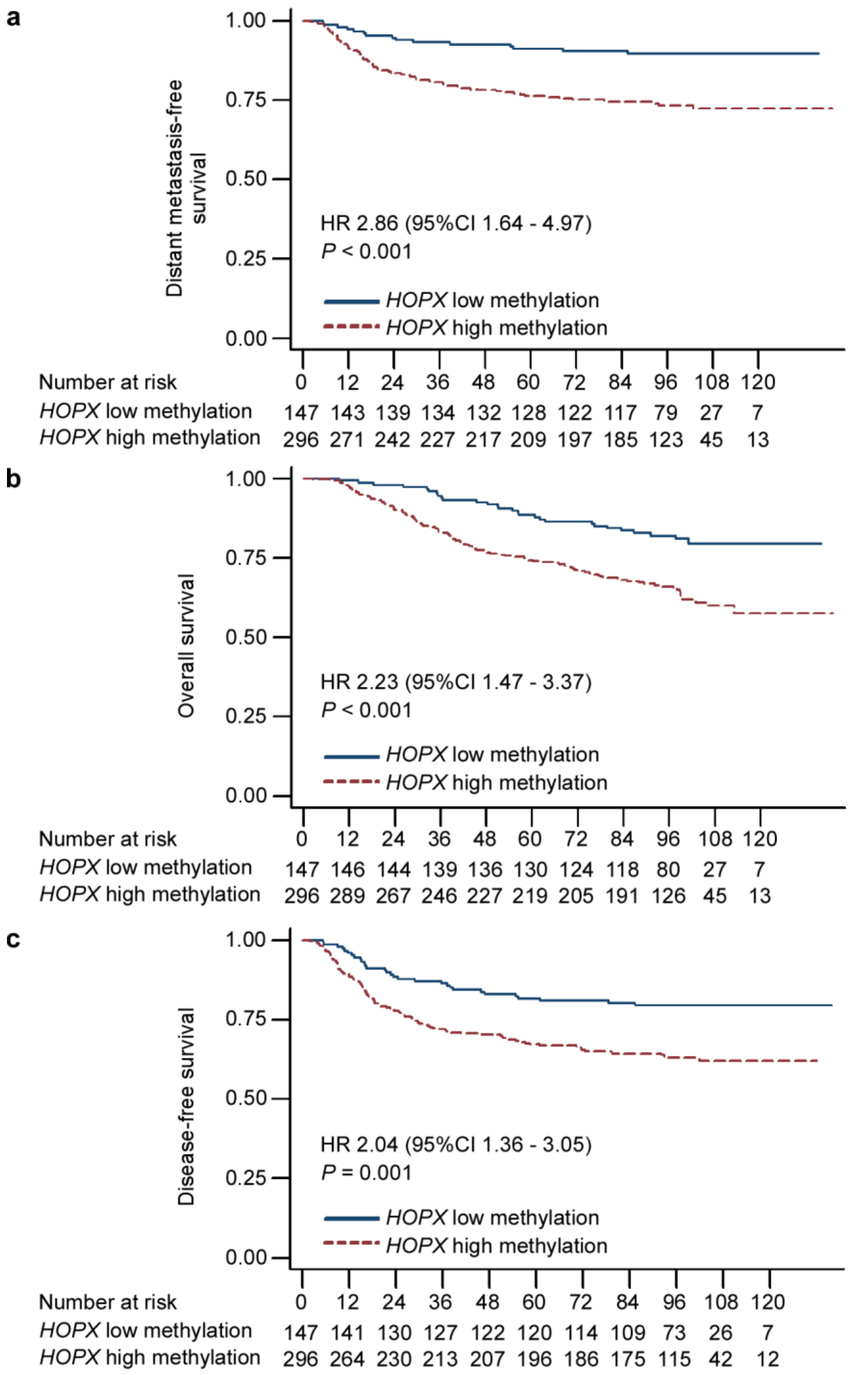

Supplementary Figure 13. *HOPX* hypermethylation is associated with poor prognosis in the combined cohort of NPC. All patients in the training and validation cohorts were combined (n = 443). **(a-c)** Kaplan-Meier analysis was performed to determine the DMFS **(a)**, OS **(b)** and DFS **(c)** according to *HOPX* methylation levels (low methylation *vs* high methylation) in NPC patients. Adjusted univariate Cox proportional hazard models were used to calculate the HR values and *P* values.

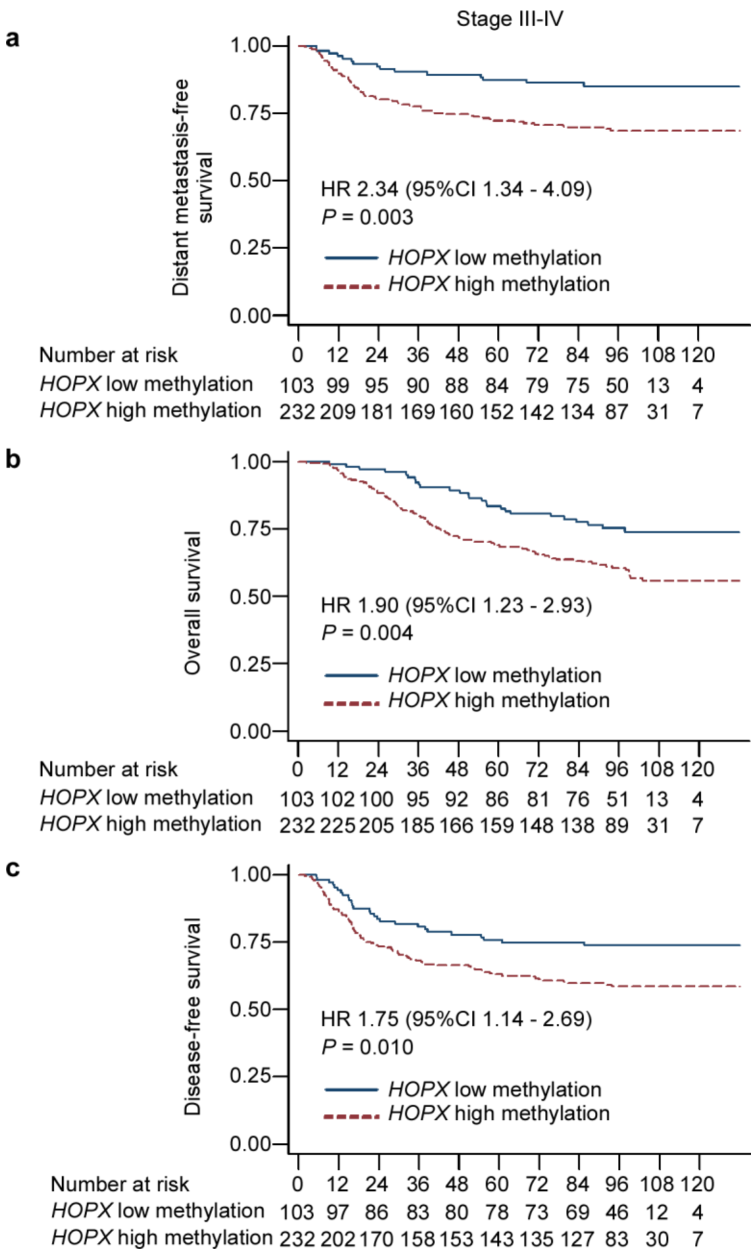

Supplementary Figure 14. *HOPX* hypermethylation is associated with poor prognosis stratified by TNM stage. (a) DMFS, **(b)** OS and **(c)** DFS in NPC patients within TNM stage III-IV (n = 335). Adjusted univariate Cox proportional hazard models were used to calculate the HR values and *P* values.

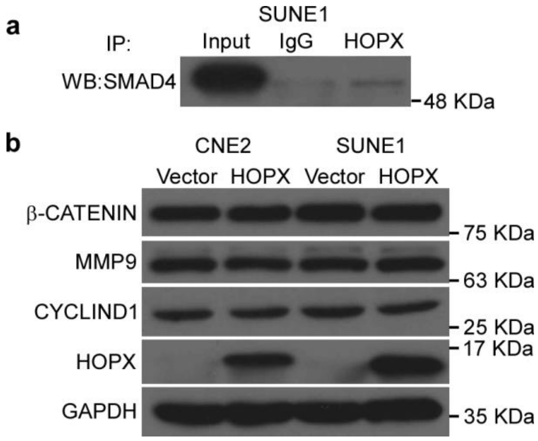

Supplementary Figure 15. HOPX exhibits little effect on the WNT signaling pathway. (a) Co-IP assay was used to measure the interaction between HOPX and SMAD4 in SUNE1 cells with stable HOPX overexpression. **(b)** Western blotting assay of HOPX, β-CATENIN, MMP9 and CYCLIND1 expression in CNE2 and SUNE1 cells with vector or HOPX overexpression. These data are representative of three independent experiments.

Supplementary Table 1. The top 10 differentially expressed genes between SUNE1 cells with HOPX or vector overexpression using tumor metastasis PCR array. *P* value is compared with the control using Student's *t*-tests; genes are ranked by *P* value. These data are representative of three independent experiments.

Gene	Functional Gene Grouping	Fold change	P value
CXCL12	Cytokines	3.26595684	0.000110047
IL1B	Cell Cycle Regulation;<br>Negative Regulation of Cell Proliferation;<br>Cytokines;<br>Apoptosis	48.6101703	0.000908618
NME4	Other Genes Related to Metastasis	37.14376425	0.001387345
MMP2	Matrix Metalloproteinases	21.40828154	0.001792716
EPHB2	Receptors	3.133358068	0.002630822
SERPINE1	Other ECM Proteins	10.77413353	0.003052426
COL4A2	Other ECM Proteins	4.537641016	0.004445524
ECADHERIN	**Cell to Cell Adhesion**	**2.213765777**	**0.004685254**
SYK	Cell to Cell Adhesion;<br>Other Genes Related to Growth	0.265441999	0.005936684
CD44	Cell to Cell Adhesion;<br>Transmembrane Receptors	3.385831498	0.006076176

Supplementary Table 2. Clinical characteristics of Nasopharyngeal Carcinoma patients according to the *HOPX* methylation level in the training and validation Cohorts.

Characteristic	Training Cohort (n = 255)			Validation Cohort (n = 188)		
	Low Methylation Group (%) n=70	High Methylation Group (%) n=185	*P* value*	Low Methylation Group (%) n=77	High Methylation Group (%) n=111	*P* value*
Age (mean ± S.D.)						
< 45	41 (58.6)	93 (50.3)	0.236	35 (45.5)	37 (33.3)	0.093
≥ 45	29 (41.4)	92 (49.7)		42 (54.5)	74 (66.7)	
Sex						
Female	17 (24.3)	52(28.1)	0.540	23 (29.9)	29 (26.1)	0.573
Male	53 (75.7)	133(71.9)		54 (70.1)	82 (73.9)	
WHO pathologic type						
I + II	1 (1.4)	5 (2.7)	0.475	8 (10.4)	6 (5.4)	0.201
III	69 (98.6)	180(97.3)		69(89.6)	105 (94.6)	
VCA-IgG						
< 80	9 (12.9)	20(10.8)	0.646	55 (71.4)	65 (58.6)	0.071
≥ 80	61 (87.2)	165(89.2)		22 (28.6)	46 (41.4)	
EA-IgG						
< 10	16 (22.9)	33(17.8)	0.364	24 (31.2)	24 (21.6)	0.104
≥ 10	54 (77.2)	152(82.2)		53 (68.8)	87 (78.4)	
TNM Stage						
I-II	23 (32.9)	38 (20.5)	**0.040**	21(27.3)	26 (23.4)	0.549
III-IV	47 (67.1)	147 (79.5)		56 (72.7)	85(76.6)	
Relapses or deaths						
No	57 (81.4)	120 (64.9)	**0.010**	60 (77.9)	67 (60.4)	**0.011**
Yes	13 (18.6)	65 (35.1)		17 (22.1)	44 (39.6)	

Distant metastasis						
No	63 (90.0)	137 (74.1)	**0.006**	69 (89.6)	82 (73.9)	**0.008**
Yes	7 (10.0)	48 (25.9)		8 (10.4)	29 (26.1)	
Death						
No	56 (80.0)	114 (61.6)	**0.011**	63 (81.8)	73 (65.8)	**0.016**
Yes	14 (20.0)	71 (38.4)		14 (18.2)	38 (34.2)	

*χ^2 test or Fisher's exact test; TNM, tumor-node-metastasis; VCA-IgA, viral capsid antigen immunoglobulin A; EA-IgA, early antigen immunoglobulin A; All patients were restaged according to the 7th edition of the AJCC Cancer Staging Manual.

Supplementary Table 3. Univariate and multivariate Cox regression analysis of *HOPX* methylation level and survival.

	Univariate analysis			Multivariate analysis		
Variable	HR	95%CI	*P* value	HR	95%CI	*P* value
**Distant metastasis-free survival**						
**Training set**						
HOPX methylation						
(Low *vs* High)	2.93	1.33-6.48	**0.008**	2.44	1.10-5.43	**0.028**
TNM stage (I-II*vs* III-IV)	6.43	2.01-20.59	**0.002**	5.00	1.55-16.10	**0.007**
Sex (Female *vs* Man)	2.05	1.00-4.19	0.049			
Age (≤ 45 *vs* > 45 years)	3.09	1.72-5.53	<0.001	2.60	1.45-4.67	0.001
WHO type (I + II *vs* III)	0.23	0.08-0.63	0.004	0.30	0.11-0.86	0.025
VCA-IgG (< 80 *vs* ≥ 80)	2.46	0.77-7.88	0.129			
EA-IgG (< 10 *vs* ≥ 10)	1.76	0.80-3.90	0.161			
**Validation set**						
HOPX methylation						
(Low *vs* High)	2.82	1.29-6.17	**0.009**	2.75	1.26-6.03	**0.011**
TNM stage (I-II *vs* III-IV)	3.11	1.10-8.79	**0.032**	3.02	1.07-8.52	**0.037**
Sex (Female *vs* Man)	1.72	0.75-3.91	0.198			
Age (≤ 45 *vs* > 45 years)	2.02	0.95-4.27	0.068			
WHO type (I + II *vs* III)	1.41	0.34-5.85	0.639			
VCA-IgG (< 80 *vs* ≥ 80)	0.70	0.35-1.43	0.329			
EA-IgG (< 10 *vs* ≥ 10)	0.72	0.36-1.44	0.357			
**Overall survival**						
**Training set**						
HOPX methylation						
(Low *vs* High)	2.25	1.27-3.99	**0.006**	2.01	1.13-3.57	**0.018**
TNM stage (I-II*vs* III-IV)	2.98	1.54-5.77	**0.001**	2.50	1.29-4.86	**0.007**
Sex (Female *vs* Man)	1.95	1.12-3.42	0.019	1.87	1.07-3.28	0.028

Age (≤ 45 *vs* > 45 years)	2.84	1.80-4.49	<0.001	2.58	1.63-4.08	<0.001
WHO type (I + II *vs* III)	0.33	0.12-0.92	0.033			
VCA-IgG (< 80 *vs* ≥ 80)	2.41	0.97-5.94	0.057			
EA-IgG (< 10 *vs* ≥ 10)	1.64	0.89-3.02	0.112			
**Validation set**						
HOPX methylation						
(Low *vs* High)	2.17	1.18-4.01	**0.013**	2.13	1.15-3.93	**0.016**
TNM stage (I-II *vs* III-IV)	3.68	1.46-9.26	**0.006**	3.61	1.43-9.08	**0.006**
Sex (Female *vs* Man)	1.34	0.70-2.55	0.379			
Age (≤ 45 *vs* > 45 years)	1.34	0.75-2.39	0.327			
WHO type (I + II *vs* III)	0.98	0.35-2.71	0.966			
VCA-IgG (< 80 *vs* ≥ 80)	0.81	0.45-1.45	0.475			
EA-IgG (< 10 *vs* ≥ 10)	0.97	0.53-1.79	0.924			
**Disease-free survival**						
**Training set**						
HOPX methylation						
(Low *vs* High)	2.15	1.19-3.91	**0.012**	1.99	1.10-3.63	**0.024**
TNM stage (I-II *vs* III-IV)	2.55	1.31-4.95	**0.006**	2.16	1.11-4.22	**0.023**
Sex (Female *vs* Man)	1.68	0.96-2.96	0.070			
Age (≤ 45 *vs* > 45 years)	2.28	1.44-3.63	<0.001	2.10	1.32-3.35	0.002
WHO type (I + II *vs* III)	0.33	0.12-0.88	0.027			
VCA-IgG (< 80 *vs* ≥ 80)	2.14	0.87-5.30	0.099			
EA-IgG (< 10 *vs* ≥ 10)	1.30	0.72-2.36	0.386			
**Validation set**						
HOPX methylation						
(Low *vs* High)	2.03	1.16-3.55	**0.013**	1.96	1.12-3.43	**0.019**
TNM stage (I-II *vs* III-IV)	3.06	1.39-6.73	**0.005**	2.97	1.35-6.53	**0.007**
Sex (Female *vs* Man)	1.49	0.81-2.75	0.202			
Age (≤ 45 *vs* > 45 years)	1.62	0.93-2.81	0.086			

WHO type (I + II *vs* III)	1.63	0.51-5.19	0.411
VCA-IgG (< 80 *vs* ≥ 80)	0.70	0.35-1.43	0.329
EA-IgG (< 10 *vs* ≥ 10)	0.89	0.51-1.56	0.679

Adjusted multivariate Cox proportional hazard models were used to calculate the HR values and P values, including *HOPX* methylation level (low *vs* high), TNM stage (stage I-II *vs* III-IV), sex (female *vs* male), age (< 45 *vs* ≥ 45 years), WHO pathological type (I + II *vs* III), VCA-IgA (< 1:80 *vs* ≥ 1:80) and EA-IgA (< 1:10 *vs* ≥ 1:10). Variables which were significantly associated with survival are presented.

Supplementary Table 4. Multivariate Cox regression analysis of *HOPX* methylation level and survival in the combined cohort.

Variable	HR	95%CI	*P* value
**Distant metastasis-free survival**			
HOPX methylation (Low *vs* High)	2.52	1.45-4.38	**0.001**
TNM stage (I-II *vs* III-IV)	3.83	1.77-8.31	**0.001**
Age (≤ 45 *vs* > 45 years)	2.30	1.45-3.65	**<0.001**
**Overall survival**			
HOPX methylation (Low *vs* High)	2.03	1.34-3.08	**0.001**
TNM stage (I-II *vs* III-IV)	2.80	1.64-4.81	**<0.001**
Age (≤ 45 *vs* > 45 years)	1.95	1.36-2.80	**<0.001**
**Disease-free survival**			
HOPX methylation (Low *vs* High)	1.84	1.23-2.77	**0.003**
TNM stage (I-II *vs* III-IV)	2.44	1.46-4.06	**0.001**
Age (≤ 45 *vs* > 45 years)	1.85	1.29-2.63	**0.001**

Adjusted multivariate Cox proportional hazard models were used to calculate the HR values and P values, including *HOPX* methylation level (low *vs* high), TNM stage (stage I-II *vs* III-IV), sex (female *vs* male), age (< 45 *vs* ≥ 45 years), WHO pathological type (I + II *vs* III), VCA-IgA (< 1:80 *vs* ≥ 1:80) and EA-IgA (< 1:10 *vs* ≥ 1:10). Variables which were significantly associated with survival are presented.

Supplementary Table 5. Primers used in this study.

Gene	Sequence (5' to 3')
**BSP PCR primers**	
HOPX-F	GTTCTTAGGGATTTTTTGTAGGAATTG
HOPX-R	CTTCCTTCACTCCTTCCTTAAAAC（5’Biotin）
**BSP sequencing primer**	
HOPX	GGATTTTTTGTAGGAATTGTAT
**Real time RT-PCR primers**	
HOPX-F	CACCACGCTGTGCCTCAT
HOPX-R	CCATTTCTGGGTCTCCTCC
SNAIL-F	TAATCCAGAGTTTACCTTCCAGC
SNAIL-R	CTCATCTGACAGGGAGGTCAG
SLUG-F	CCCTGAAGATGCATATTCGGA
SLUG-R	CTGCAAATGCTCTGTTGCAG
ZEB1-F	TCACTAGTGTTTACCAGAACAGTG
ZEB1-R	GAACACTGTTCTGGTCAGCA
ZEB2-F	CTACAAGCGCTTGACATCAC
ZEB2-R	TAGCATTTGGTGCTGATCTGTC
TWIST1-F	AGTCTTACGAGGAGCTGCAG
TWIST1-R	CTCTGGAGGACCTGGTAGAG
FOXC2-F	CTTCTACCGGGAGAACAAGC
FOXC2-R	CTCCTTCTCCTTGGACACGT
ECADHERIN-F	GAAGAGGACCAGGACTTTGAC
ECADHERIN-R	GTAGTCATAGTCCTGGTCTTTGTC
α-CATENIN-F	CCTGAGGAGTTGGATGACTC
α-CATENIN-R	TCCTTTACCTCGGGTAAAGTC

VIMENTIN-F	ATTCCACTTTGCGTTCAAGG
VIMENTIN-R	CTTCAGAGAGAGGAAGCCGA
FIBRONECTIN-F	CACGATGATATGGAGAGCCA
FIBRONECTIN-R	TATTTGGTGGCCACCATAAGTC
SRF-F	CACCATCATGACGTCATCCG
SRF-R	CTATCACAGCCATCTGGTGG
**siRNA sequences**	
siRNA-NC-F	5'-UUCUCCGAACGUGUCACGUTT-3'
siRNA-NC-R	5'-ACGUGACACGUUCGGAGAATT-3'
si*HOPX*-1-F	5'-GAAGCUAUGUGUAUCUUCUTT-3'
si*HOPX*-1-R	5'-AGAAGAUACACAUAGCUUCTT-3'
si*HOPX*-2-F	5'-CCCUAAGUCACUUUCCUUATT-3'
si*HOPX*-2-R	5'-UAAGGAAAGUGACUUAGGGTT-3'
**ChIP-PCR primers**	
SNAIL-1-F	AGGAGTCCCCGCCCGGGCTCTCACC
SNAIL-1-R	GCAGCAGCGCCGCCAACTCCCTTAA
SNAIL-2-F	GGGGCGTCAGAAGCGCTCAGACCAC
SNAIL-2-R	GTTCGCTGGCGCAGCGCGGGTCGTC
SNAIL-3-F	GTGTCCCTTTCCTCGCTTCCTC
SNAIL-3-R	CGGGACACCTGACCTTCCGAC
SNAIL-4-F	TTCTGTCCGGGGCTGTGCCCTG
SNAIL-4-R	CGCACGTGGCTCTCGGCGGCTTG
SNAIL-5-F	AGTCACCCCGACCCCCTGTCAG
SNAIL-5-R	CACTCAGAGCCTCTCCCGAAG
SNAIL-6-F	TCCAAACTCCTACGAGGCCCTG
SNAIL-6-R	TCCCAGAGGAAGTGAAGAATTA

Supplementary Table 6. Antibodies used in this study.

Antibody	Company	Catalog no.	Dilution
**Western blotting**			
GAPDH	Santa Cruz	sc-25778	1:1000
HOPX	Santa Cruz	SC-30216	1:200
ECADHERIN	BD	610181	1:2000
a-CATENIN	BD	610193	1:500
VIMENTIN	Proteintech	10366-1-AP	1:1000
FIBRONECTIN	Proteintech	156131-AP	1:500
SNAIL	Abcam	ab180714	1:1000
SRF	Proteintech	16821-1-AP	1:800
HDAC2	Proteintech	12922-3-AP	1:1000
SMAD4	Proteintech	10231-1-AP	1:200
β-CATENIN	Proteintech	51067-2-AP	1:500
CYCLIND1	Proteintech	60186-1-Ig	1:500
MMP9	Proteintech	10375-2-AP	1:500
Mouse	CST	7076	1:5000
Rabbit	CST	7074	1:5000
**Immunohistochemistry**			
HOPX	Santa Cruz	SC-30216	1:100
SNAIL	Abcam	ab180714	1:200
**Immunofluorescence**			
SRF	Proteintech	16821-1-AP	1:100
HDAC2	Santa Cruz	SC-6296	1:100
HOPX	Santa Cruz	SC-30216	1:100
ECADHERIN	BD	610181	1:100
a-CATENIN	BD	610193	1:100

VIMENTIN	Proteintech	10366-1-AP	1:100
goat Anti-rabbit, Alexa Fluor® 594 IgG secondary antibody	Life	A-11008	1:1000
goat Anti-mouse, Alexa Fluor® 594 IgG secondary antibody	Life	A-11001	1:1000
rabbit Anti-goat, Alexa Fluor® 647 IgG secondary antibody	Life	A-27018	1:1000
**Co-Immunoprecipitation**			
HOPX	Santa Cruz	SC-30216	3μg
SRF	Proteintech	16821-1-AP	3μg
HDAC2	Proteintech	12922-3-AP	3μg
IgG	Proteintech	30000-0-AP	3μg
**Chromatin Immunoprecipitation**			
HOPX	Santa Cruz	SC-30216	2.0 ml per 0.5 ml Protein A/G sepharose beads
HDAC2	Abcam	ab7029	0.5mg per 5ml Protein A/G sepharose beads
H3K9Ac	Abcam	ab10812	0.2mg per 2ml Protein A/G sepharose beads
SRF	CST	5147	1:100
IgG	Sigma	SLBJ3775V	1:100

Supplementary Figure 16. Full unedited western blotting gels for all figures.

Full unedited gel for Figure 2e

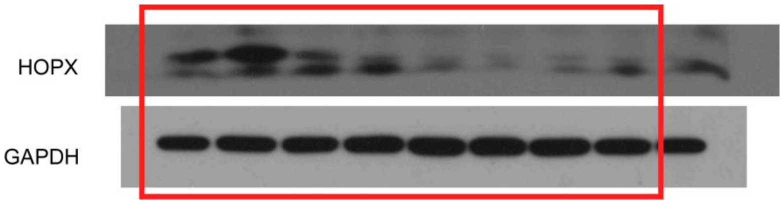

Full unedited gel for Figure 4d,e

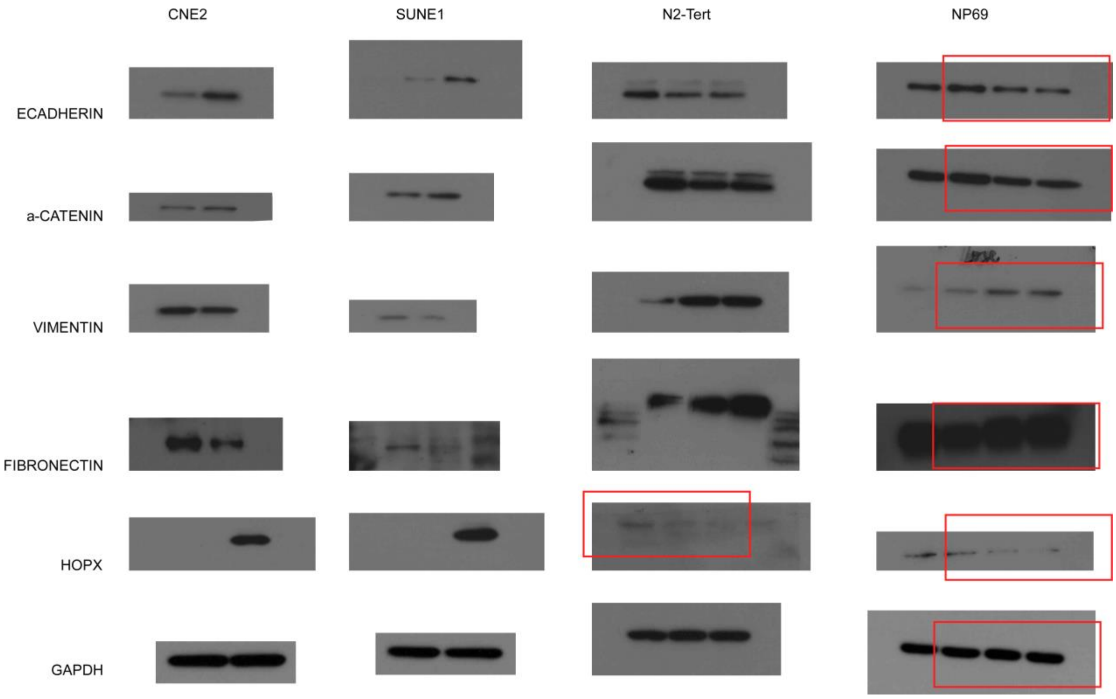

Full unedited gel for Figure 5c, d and g

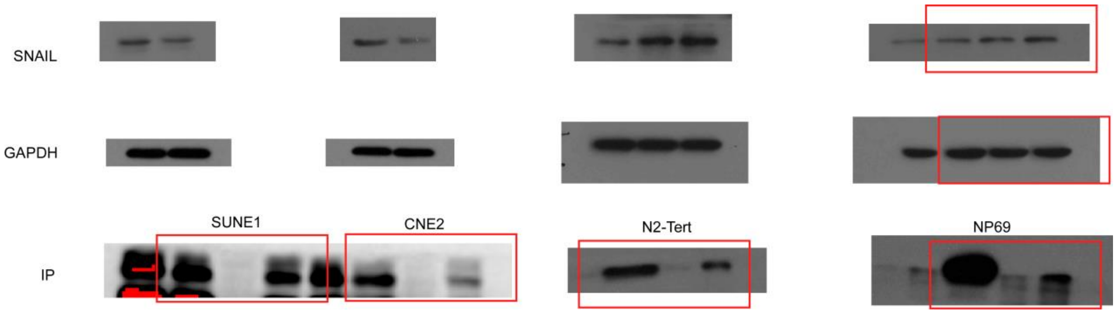

Full unedited gel for Figure 6c

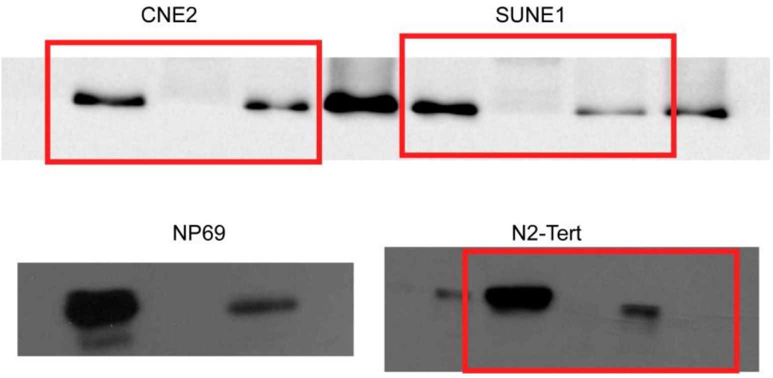

Full unedited gel for Figure 7d

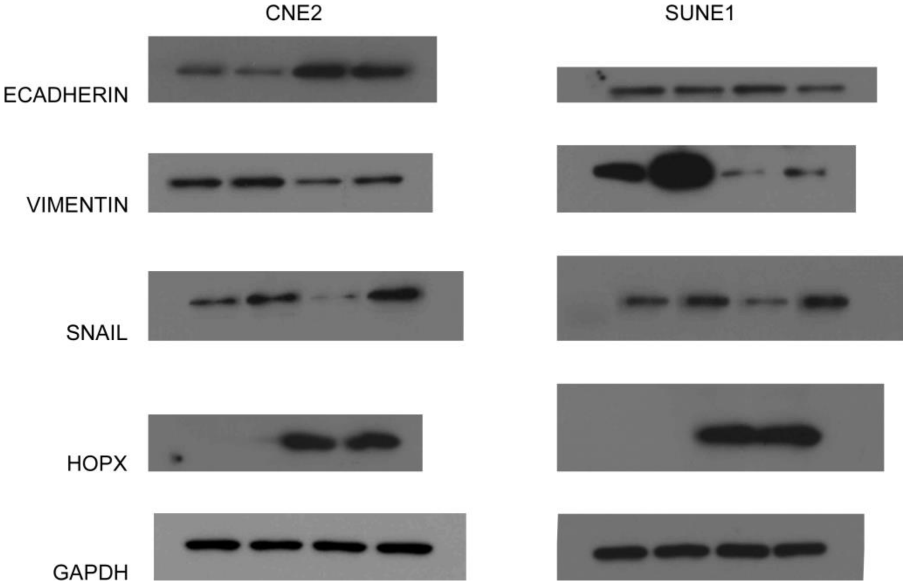

Full unedited gel for Supplementary Figure 3a

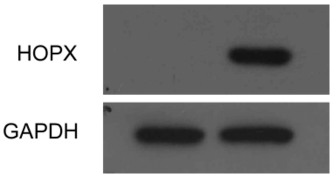

Full unedited gel for Supplementary Figure 4a

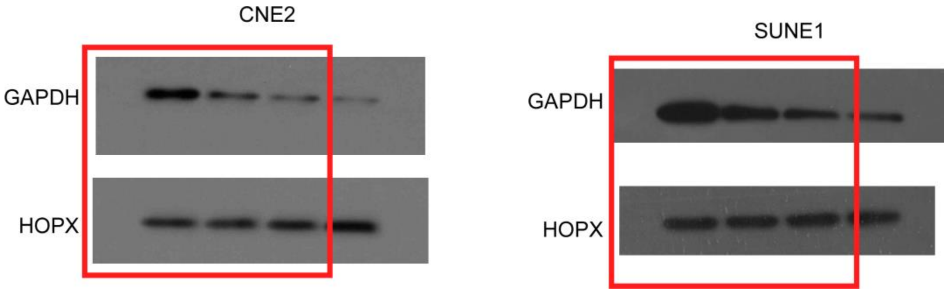

Full unedited gel for Supplementary Figure 7c

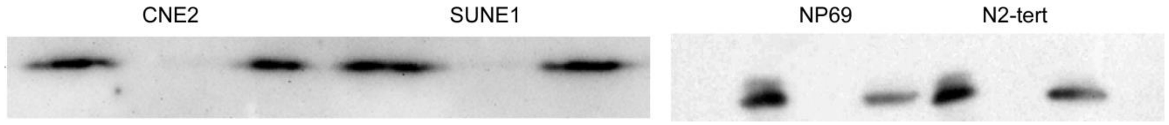

Full unedited gel for Supplementary Figure 8a,b,d

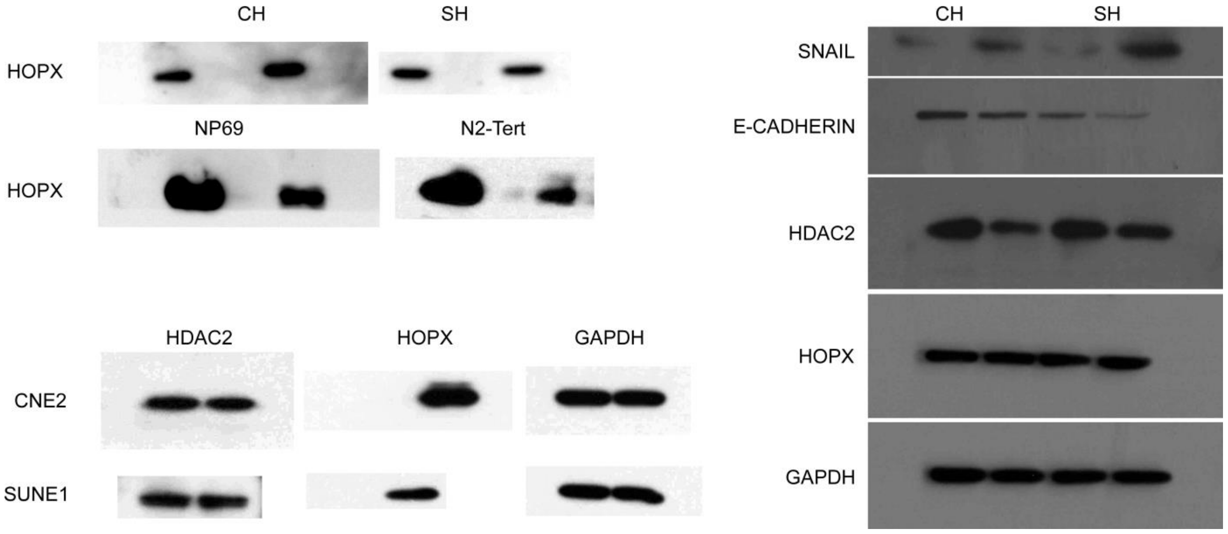

Full unedited gel for Supplementary Figure 9b,c,g

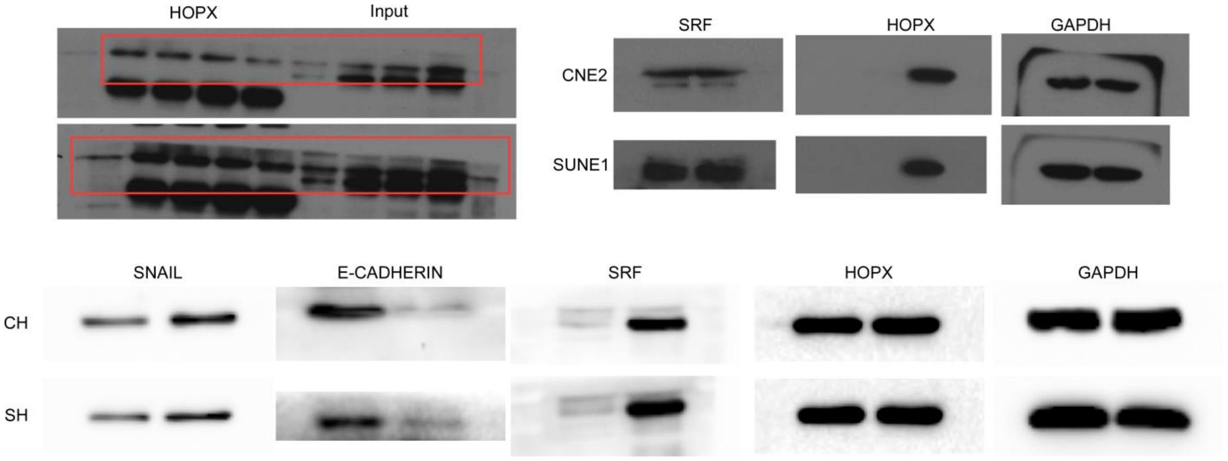

Full unedited gel for Supplementary Figure 15

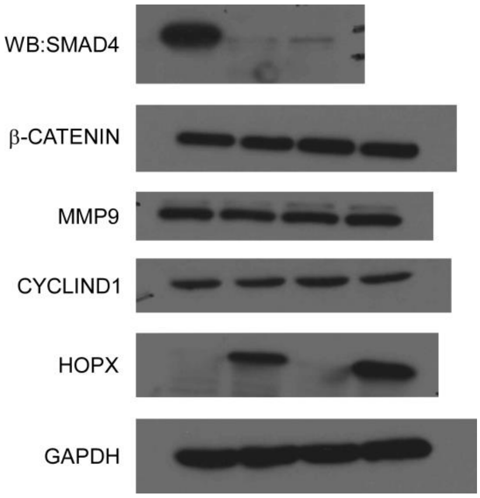

Supplement: Supplementary Information — Supplementary Figures, Supplementary Tables. [file ncomms14053-s1.pdf]
